# The bacterial microbiome of field-collected *Dermacentor marginatus* and *Dermacentor reticulatus* from Slovakia

**DOI:** 10.1186/s13071-019-3582-9

**Published:** 2019-06-27

**Authors:** Yan-Kai Zhang, Zhi-Jun Yu, Duo Wang, Víchová Bronislava, Peťko Branislav, Jing-Ze Liu

**Affiliations:** 1 Hebei Key Laboratory of Animal Physiology, Biochemistry and Molecular Biology, College of Life Sciences, Hebei Normal University, Shijiazhuang, 050024 Hebei China; 2 Institute of Parasitology, Slovak Academy of Sciences, 04001 Kosice, Slovak Republic; 3 University of Veterinary Medicine and Pharmacy in Kosice, 04185 Kosice, Slovak Republic

**Keywords:** Bacterial microbiome, *Dermacentor marginatus*, *Dermacentor reticulatus*, Slovakia

## Abstract

**Background:**

The important roles of microbial flora in tick biology and ecology have received much attention. *Dermacentor marginatus* and *Dermacentor reticulatus* are known vectors of various pathogens across Europe, including Slovakia. However, their bacterial microbiomes are poorly explored.

**Methods:**

In this study, bacterial microbiomes of field-collected *D. marginatus* and *D. reticulatus* from Slovakia were characterized using *16S* rRNA high-throughput sequencing.

**Results:**

Different analyses demonstrated that the *D. marginatus* and *D. reticulatus* microbiomes differ in their diversity and taxonomic structures. Furthermore, species- and sex-specific bacteria were detected in the two species. A possible bacterial pathogen “*Candidatus* Rhabdochlamydia sp.” was detected from *D. marginatus* males. Among the observed bacteria, *Rickettsia* showed high abundance in the two species. Several maternally inherited bacteria such as *Coxiella*, *Arsenophonus*, *Spiroplasma*, *Francisella* and *Rickettsiella*, were abundant, and their relative abundance varied depending on tick species and sex, suggesting their biological roles in the two species.

**Conclusions:**

The bacterial microbiomes of field-collected *D. marginatus* and *D. reticulatus* were shaped by tick phylogeny and sex. Maternally inherited bacteria were abundant in the two species. These findings are valuable for understanding tick-bacteria interactions, biology and vector competence of ticks.

**Electronic supplementary material:**

The online version of this article (10.1186/s13071-019-3582-9) contains supplementary material, which is available to authorized users.

## Background

Ticks are obligate blood-sucking parasitic arthropods, feeding on mammals, reptiles, birds and amphibians. More than 900 tick species have been identified worldwide, and many species are of great economic and epidemiological importance [1, 2]. Ticks carry and transmit various pathogens, including bacteria, viruses, protozoans and helminths [3]. Tick-borne diseases (TBDs) caused by these pathogens, such as human granulocytic anaplasmosis (HGA), Lyme disease, tick-borne encephalitis (TBE), babesiosis, etc., are distributed worldwide and resulting in serious harms [4]. Globally, tick-borne pathogens cause over 100,000 cases of human diseases yearly [1]. Every year about 65,000 people are infected in EU countries (https://ecdc.europa.eu/en/tick-borne-diseases). Habitat changes, climate changes, human activities and globalization are responsible for the emergence, spreading and migration of hosts, vectors, parasites and pathogens as well as for the rising incidence and diversity of vector-borne infections [5–7]. To date, at least 33 new tick-borne pathogens (TBPs) have been found in China [8]. Similarly, in Europe, climate change most predominantly affects seasonal range expansions and contractions of vector-borne diseases even in small areas [9]. For example, TBE cases moved from lowlands to sub mountainous areas in Slovakia since 1980, most likely because of rising temperature [10].

Given the importance of ticks as vectors of pathogens, aspects of tick biology and ecology have received much attention [2, 11, 12]. The tick microbiome comprises of communities of TBPs, viruses, bacteria and eukaryotes [13]. The rapid development of DNA and RNA sequencing platforms, especially high-throughput next-generation sequencing (NGS) technologies, have served as key drivers in our ability to realize the complexity of the tick microbiome in great detail [13, 14]. A series of studies have suggested that these non-pathogenic microorganisms are also abundant in ticks and have important roles in affecting tick biology and pathogen transmission [4, 13, 15–19]. A typical example is *Coxiella*-like endosymbiont, which has been reported as essential for tick survival and reproduction in *Amblyomma americanum* [20], *Haemaphysalis longicornis* [21] and *Rhipicephalus microplus* [22]. Recently, empirical evidence of an obligate B vitamin provisioning symbiont in ticks was found [23]. Non-pathogenic microorganisms also influence pathogens in different ways. For example, *Ixodes scapularis* fed on antibiotic-treated mice exhibited a modified gut microbiome, resulting in increased feeding and low *Borrelia burgdorferi* colonization rates [24]. Similarly, Gall et al. [15] found that a disrupted microbiome of *Dermacentor andersoni* is correlated with *Anaplasma marginale* and *Francisella novicida* susceptibility. These findings are paramount to fully exploiting the microbiome in order to control ticks and TBDs.

*Dermacentor marginatus* and *Dermacentor reticulatus* are two key tick vectors of various pathogens [1, 25, 26]. They are widespread in Europe, ranging from Portugal to Ukraine (and continue to the east of Kazakhstan) [27]. They are also distributed in China [25] and Russia [28]. Slovakia is located in central Europe; its climate lies between the temperate and continental climate zones with relatively warm summers and cold, cloudy and humid winters. The distributions and vector competences of *D. marginatus* and *D. reticulatus* have been fully investigated in Slovakia [29–37]. A survey found that *D. reticulatus* has extended its range in the surroundings of its former habitats [31]. In addition, the influences of global climate changes on the structures and dynamics of TBDs in mountain areas were assessed under a research project supported by governments of China and Slovakia.

It is evident that *D. marginatus* and *D. reticulatus* have great importance in medical and animal husbandry in Slovakia. Investigation of their microbiomes will aid in the control of ticks and TBDs. In this study, bacterial microbiomes of field-collected *D. marginatus* and *D. reticulatus* from Slovakia were characterized using *16S* rRNA high-throughput sequencing.

## Methods

### Tick collection and sample preparation

*Dermacentor marginatus* and *D. reticulatus* were collected in the area of Slovak Karst, which is one of the mountain ranges of the Slovenské Rudohorie Mountains in the Carpathians in southern Slovakia. It consists of a complex of huge karst plains and plateaus. The area has been a protected landscape area since 1973, and in 2002, the Slovak Karst National Park was established. The park is also a UNESCO Biosphere Reserve and forms a UNESCO World Heritage site. Several endemic species of animals and plants live in this region, which has warm and moderately humid climate [38]. Tick collection sites were established in a small area situated on the northern grassy slope covered with scattered islands of xerophilous shrubs (212 meters above sea level, 48° 34′53.88″ N, 20° 46′ 44.43″ E), near the Hrhov village in eastern Slovakia. A small river and lake to the north and an old oak and hornbeam forest to the south surround the sampling site. These areas are usually used as the pastures for livestock grazing. Ticks were collected by the standard flagging method in the early spring of 2017 and were identified into developmental stages, species and sex using the taxonomic key [39]. Before study, ticks were stored at − 80 °C. A total of 48 adult ticks were used for this study (*D. marginatus*, *n* = 24; *D. reticulatus*, *n* = 24). For each species, according to tick sex, samples were grouped into three pools of 4 individuals each, and sample names are shown in Table 1.

**Table 1:** Information of tick samples used for bacterial microbiome analysis

Sample name	Tick species	Sex	*n*
DmarF1	*Dermacentor marginatus*	Female	4
DmarF2		Female	4
DmarF3		Female	4
DmarM1		Male	4
DmarM2		Male	4
DmarM3		Male	4
DretF1	*Dermacentor reticulatus*	Female	4
DretF2		Female	4
DretF3		Female	4
DretM1		Male	4
DretM2		Male	4
DretM3		Male	4

*Abbreviation*: n, number of individuals

### DNA extraction

Prior to DNA extraction, ticks were surface-sterilized in three washes of 70% ethanol followed by one wash of sterile, nuclease-free, deionized water to avoid contamination from the environment. DNA extraction was performed using a QIAamp Fast DNA Stool Mini Kit (Qiagen, Hilden, Germany). The concentration and quality of DNA was measured using a Nanodrop 2000 (Thermo Fisher Scientific, Wilmington, DE, USA) and 1% gel electrophoresis detection, respectively.

### *16S* rRNA PCR amplification and sequencing

The V3-V4 region of the bacterial *16S* ribosomal RNA (rRNA) gene was amplified by PCR with barcode-indexed primers (338F: 5′-ACT CCT ACG GGA GGC AGC AG-3′ and 806R: 5′-GGA CTA CHV GGG TWT CTA AT-3′), using TransStart Fastpfu DNA Polymerase (TransGen, Beijing, China). PCRs were performed on GeneAmp® 9700 PCR instrument (Applied Biosystems, Foster City, CA, USA). This primer set, resulted in 420- to 460-bp PCR products. Amplicons were then purified by gel extraction (AxyPrep DNA GelExtraction Kit; Axygen Biosciences, Union City, CA, USA) and were quantified using QuantiFluor-ST (Promega, Madison, WI, USA). The purified amplicons were pooled in equimolar concentrations, and paired-end sequencing was performed on an Illumina MiSeq PE300 platform (Shanghai Majorbio Bio-pharm Technology Co., Ltd, Shanghai, China) using standard protocols.

### Data analysis

The data were analyzed on the free online platform of Majorbio I-Sanger Cloud Platform (http://www.i-sanger.com). MiSeq sequence data were merged and filtered using the Trimmomatic software as previously described [40]. Quality-filtered merged reads were aligned to the Silva database [41] and screened for chimeras using Uchime algorithm [42]. Sequences with 97% similarity were then grouped into operational taxonomic units (OTUs) using OptiClust clustering algorithm [43]. The OTU table was processed in Qiime (MacQIIME v.1.9.0) [44]. OTUs were taxonomically assigned using the RDP Classifier v.2.2 [45] against the Greengenes *16S* rRNA database v.13.5 with 70% confidence [46], and relative OTU abundances were summarized across taxonomic levels from domain to species.

Sufficient sequencing depth was determined based on rarefaction curves for observed number of OTUs from all samples. The bacterial composition of each sample was visualized as a bar figure. Sobs’ index and Shannon’s diversity index were calculated to measure bacterial community richness and diversity between groups, and Student’s t-test was used to test whether the two indices are significantly different. Analysis of similarities (ANOSIM) was used, with 999 permutations based on the Bray-Curtis index, to determine the percent variation of bacterial composition explained by tick species and sex. Beta diversity was examined using weighted and unweighted UniFrac analysis to compare the different groups and plotted in a principal coordinate analysis (PCoA). Wilcoxon rank-sum test was used to test for differences of bacterial composition between tick species and between males and females.

## Results

### MiSeq sequencing data

A total of 12 pooled samples were sequenced (Table 1), resulting in 1,045,584 raw reads. After filtration, 522,792 reads were generated and taxonomically assigned. The number of reads per sample was 30,632 to 65,290 (Additional file 1: Table S1). Rarefaction curves of the Shannonʼs index at OTU level indicated sufficient sequencing coverage, as demonstrated by observed Shannonʼs index accumulation curves reaching a plateau (Additional file 2: Figure S1).

### Bacterial microbiome composition

**Fig. 1:**
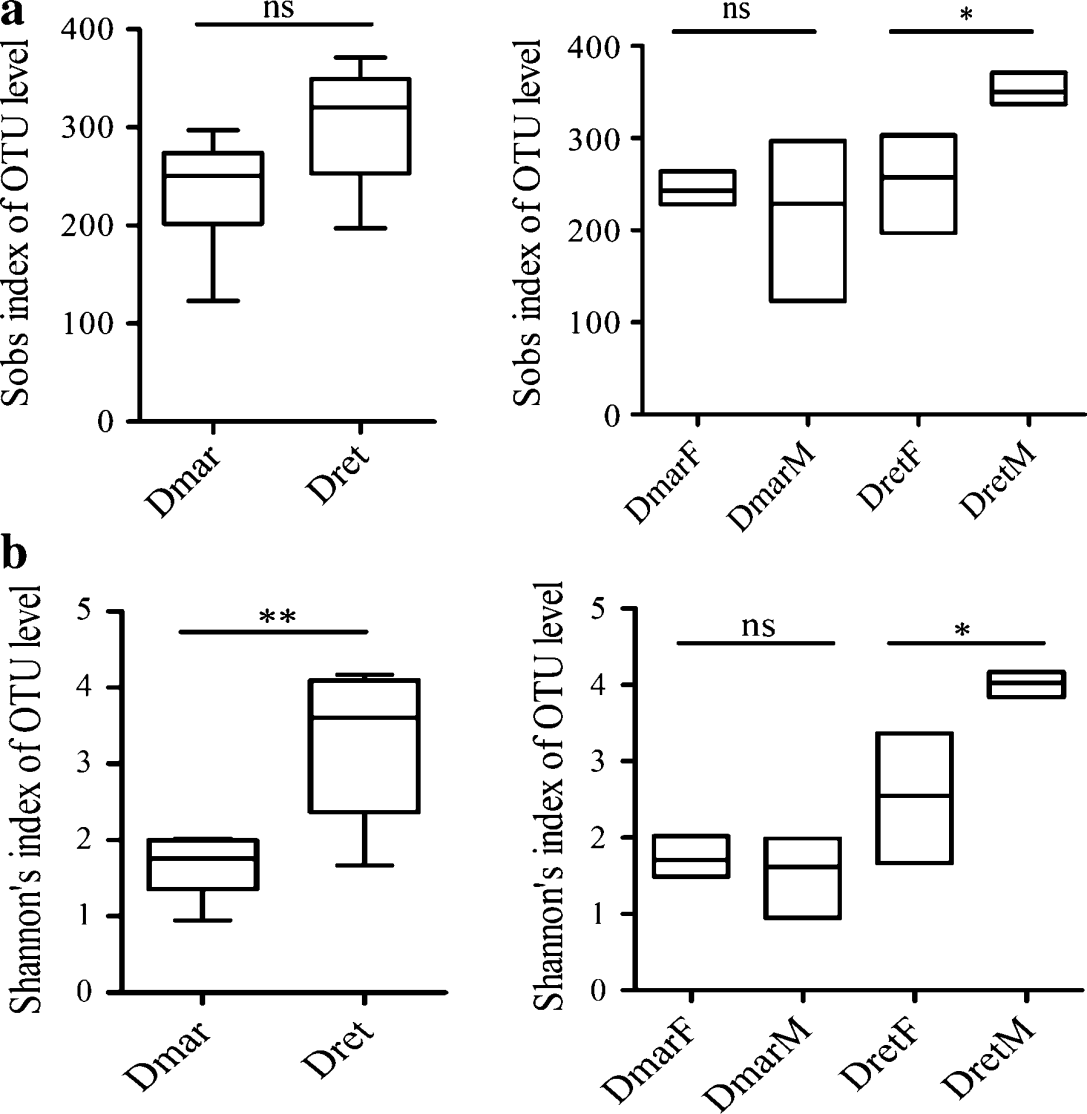
Alpha diversity of bacterial microbiomes in *Dermacentor marginatus* and *Dermacentor reticulatus.* **a** Sobs index for each sample group. **b** Shannonʼs index for each sample group. *P < 0.05, **P < 0.01; ns indicates that differences between sample groups are not significant. *Abbreviations*: DmarF, female *D. marginatus*; DmarM, male *D. marginatus*; DretF, female *D. reticulatus*; DretM, male *D. reticulatus*

In total, 550 OTUs were detected in 12 samples (Additional file 3: Table S2). The richness of the bacterial microbiome in *D. reticulatus* was higher than in *D. marginatus*, but the difference was not significant (*t*_(10)_ = 1.1913, *P* = 0.084) (Fig. 1a). The diversity of the bacterial microbiome in *D. reticulatus* was significantly higher than in *D. marginatus* (*t*_(10)_ = 3.757, *P* = 0.0037) (Fig. 1b). In *D. marginatus*, the bacterial microbiomes of females and males exhibited similar richness (*t*_(4)_ = 0.2622, *P* = 0.81, Fig. 1a) and diversity (*t*_(4)_ = 0.2527, *P* = 0.81, Fig. 1b) levels. However, the bacterial microbiome in male *D*. *reticulatus* had relatively higher richness (*t*_(4)_ = 2.791, *P* = 0.049, Fig. 1a) and diversity (*t*_(4)_ = 2.954, *P* = 0.042, Fig. 1b) compared with females.

**Fig. 2:**
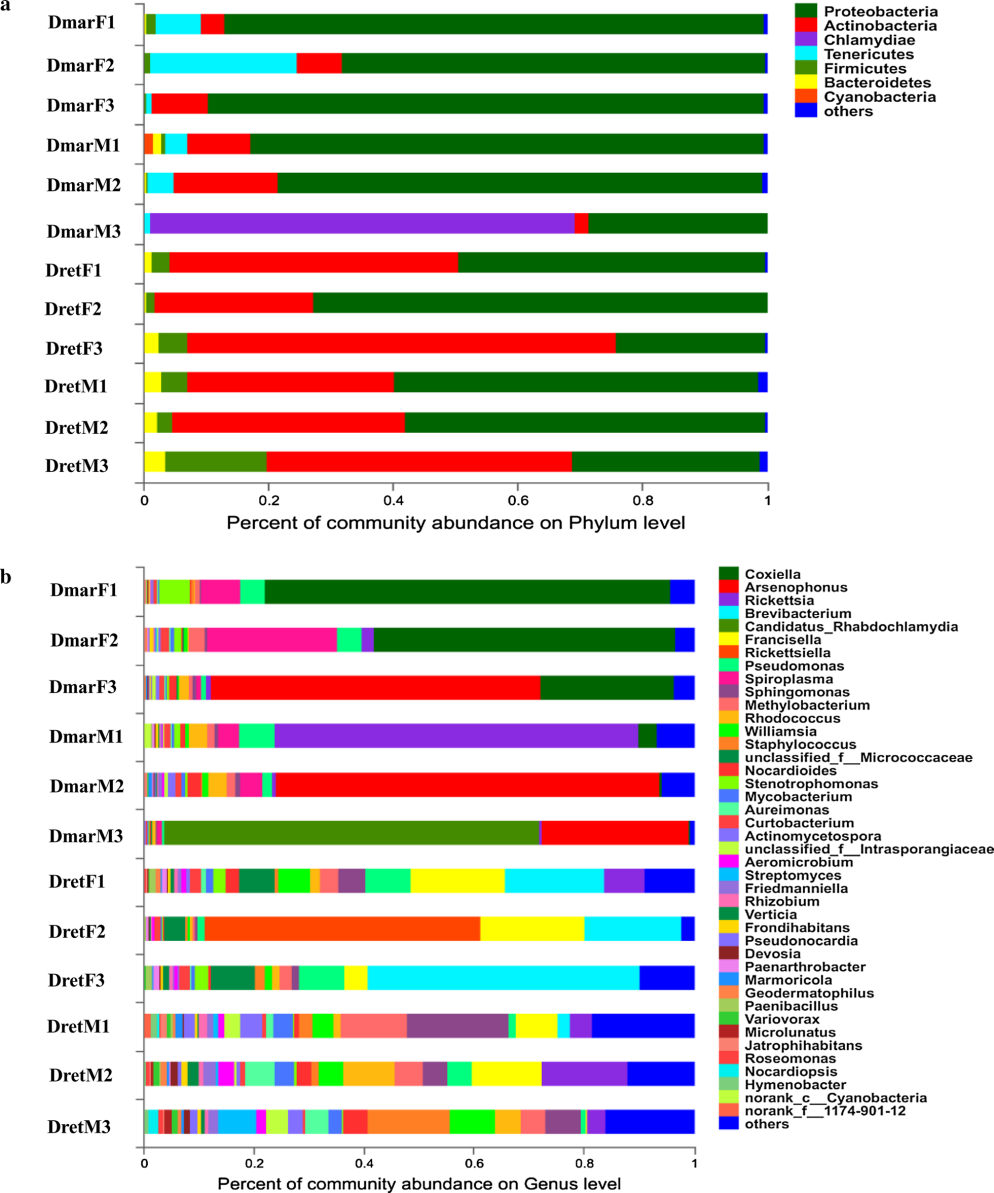
Relative abundance of bacterial phyla (**a**) and genera (**b**) in *Dermacentor marginatus* and *Dermacentor reticulatus. Abbreviations*: DmarF, female *D. marginatus*; DmarM, male *D. marginatus*; DretF, female *D. reticulatus*; DretM, male *D. reticulatus*

Bacterial microbiome was further assigned to 22 phyla, 40 classes, 89 orders, 161 families, 290 genera and 396 species. At the phylum level, Proteobacteria were the most dominant (60.4%), followed by Actinobacteria (25.76%), Chlamydiae (5.69%), Tenericutes (3.37%), Firmicutes (2.92%), Bacteroidetes (1.25%) and other phyla (0.63%). Most of bacterial phyla (17 of 22) were shared by *D. marginatus* and *D. reticulatus*, and by males and females within the same species. Chlamydiae were only found in male *D. marginatus*. Chlorobi and Parcubacteria were only detected in *D. marginatus*, while Ignavibacteriae, Armatimonadetes and an unclassified phylum were specific phyla in *D. reticulatus* (Fig. 2a, Additional file 3: Table S2). At the genus level, 218 bacterial genera were shared by *D. marginatus* and *D. reticulatus*. Among them, *Rickettsia* had the highest relative abundance (13.67%), followed by *Brevibacterium* (11.93%), “*Candidatus* Rhabdochlamydia” (9.3%), *Pseudomonas* (5.83%), *Sphingomonas* (5.36%), *Methylobacterium* (4.8%), *Rhodococcus* (3.83%) and *Williamsia* (3.68%). Of the 23 genera only detected in *D. marginatus*, *Coxiella*, *Arsenophonus* and *Spiroplasma* exhibited higher relative abundance (Fig. 2b). Two out of the 49 specific genera in *D. reticulatus*, *Francisella* and *Rickettsiella* had relatively high abundance (Fig. 2b).

**Fig. 3:**
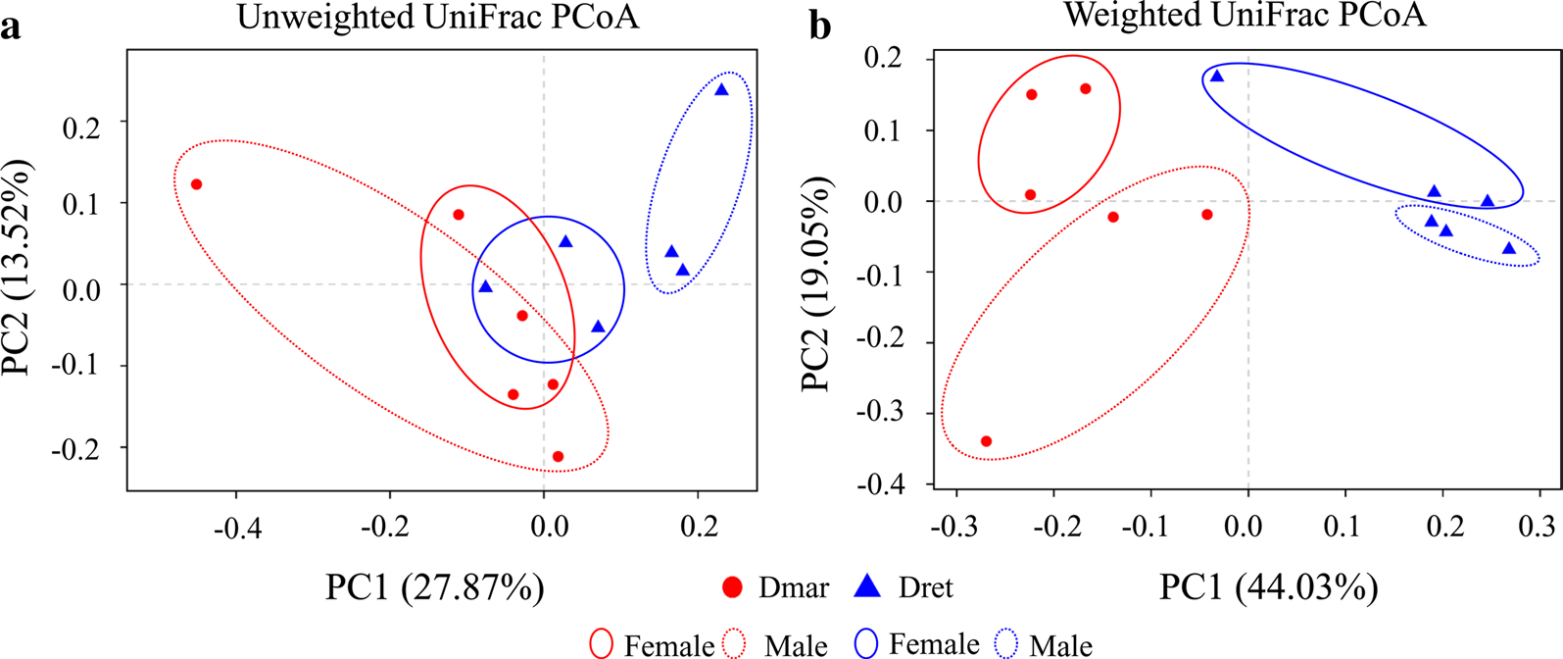
PCoA plots of unweighted UniFrac distances (**a**) and weighted UniFrac distances (**b**) of all samples. *Abbreviations*: DmarF, female *D. marginatus*; DmarM, male *D. marginatus*; DretF, female *D. reticulatus*; DretM, male *D. reticulatus*

Bacterial microbiome compositions of *D. marginatus* and *D. reticulatus* were significantly different according to ANOSIM (pseudo-*R* = 0.652, *P* = 0.003). Furthermore, PCoA analyses suggested that bacterial microbiome compositions were similar within the same tick species and the same tick sex (Fig. 3a, 3b).

### Bacterial relative abundance differences

**Fig. 4:**
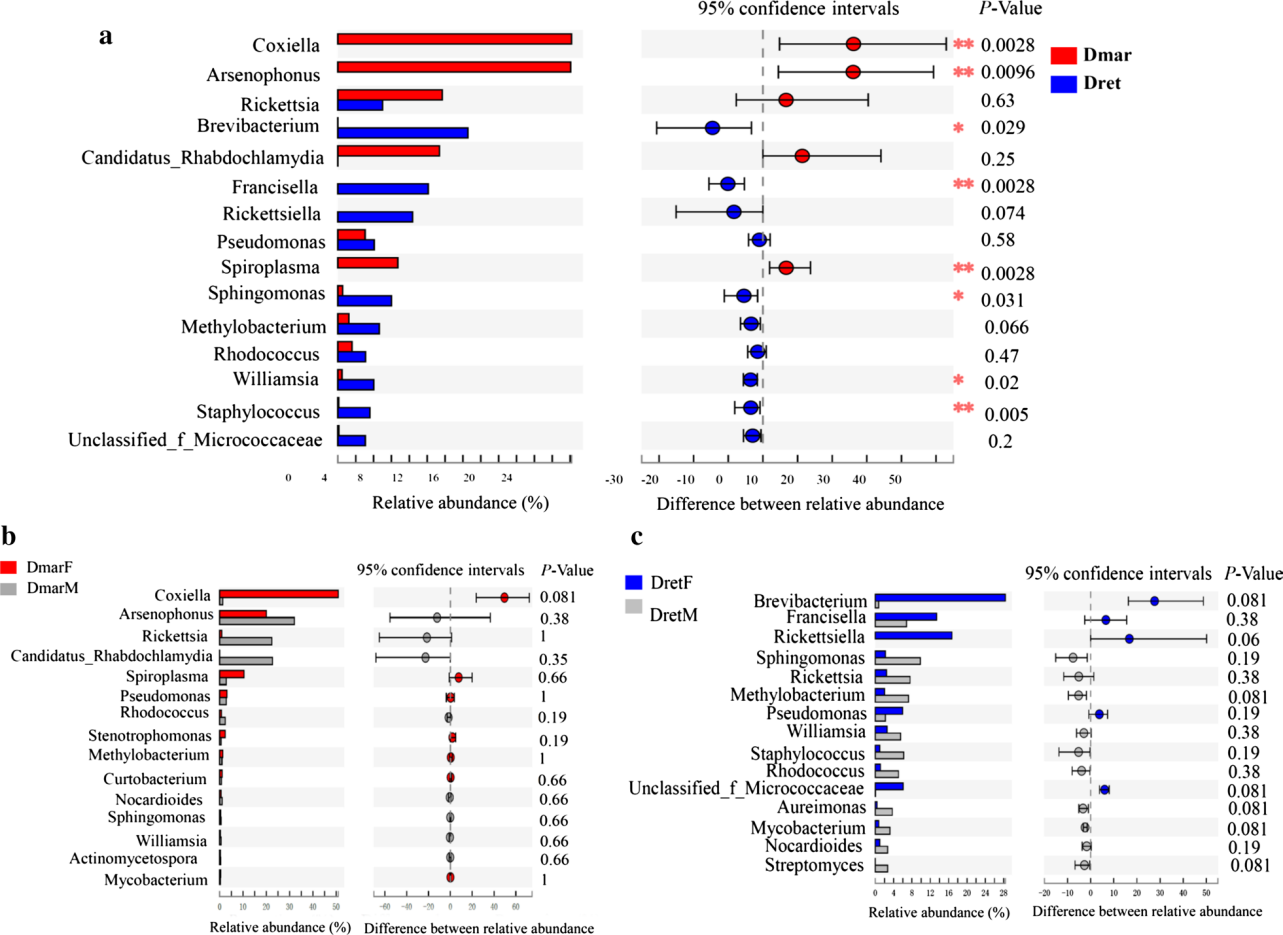
Differences of bacterial composition between tick species and between males and females. *P < 0.05, **P < 0.01. *Abbreviations*: DmarF, female *D. marginatus*; DmarM, male *D. marginatus*; DretF, female *D. reticulatus*; DretM, male *D. reticulatus*

The relative abundance of the 15 top bacterial genera was compared using the Wilcoxon rank-sum test to detect possible differences. Five genera, i.e. *Coxiella*, *Arsenophonus*, *Spiroplasma*, *Francisella* and *Rickettsiella*, were detected at higher relative abundance (ranging between 6.7–26.2%). Except for “*Candidatus* Rhabdochlamydia” and *Stenotrophomonas*, most of the remaining genera had higher abundance in *D. marginatus* than that in *D. reticulatus*, and significant differences were found in the relative abundance of *Williamsia* and *Staphylococcus* (*P* < 0.05, Fig. 4a). In *D. marginatus*, females harbored more *Coxiella*, *Spiroplasma* and *Stenotrophomonas*. However, the relative abundance of *Arsenophonus*, *Rickettsia* and “*Candidatus* Rhabdochlamydia” were relatively high in males (Fig. 4b). In *D. reticulatus*, bacterial relative abundance differences between females and males were also observed, although the differences were not significant (Fig. 4c).

## Discussion

In recent years, studies of the tick microbiome have been increasing, especially with the development and application of NGS technologies [14]. These studies have investigated the bacterial communities in different ticks [16, 47–52], and explored the influence of tick microbiomes on pathogen transmission and susceptibility [15, 16]; their findings strongly suggest that the bacterial microbiome has important roles in tick biology and ecology, and has a potential application in tick control.

To our knowledge, this study is the first to investigate the bacterial microbiomes of field-collected *D. marginatus* and *D. reticulatus* from Slovakia. The examined ticks were collected from the Slovak Karst region, of which the chosen study area (Hrhov) in particluar represents a significant biodiversity hotspot, not only in Slovakia but in the whole of central Europe. It is characterized by the presence of several endemic plant and animal species, and also by the co-occurrence of several tick species. In this area, in addition to the widely distributed *Ixodes ricinus*, tick species which are typical for the forest-steppe zones (*D. marginatus* and *Haemaphysalis inermis*) and the alluvial forests and wet meadows (*D. reticulatus* and *Haemaphysalis concina*) are also present. Moreover, the occurrence of *Ixodes frontalis* has been reported in this area [53]. Previous studies have found several pathogens in *D. marginatus* and *D. reticulatus* collected from Slovakia [34, 35, 37, 54]. In comparison, the bacterial microbiome of the two species is less known, and there are only two studies (on *D. reticulatus* in Russia [47] and *D. marginatus* in Turkey [48]).

MiSeq sequencing data generated from 12 pooled samples showed high quality and can be used for further analyses. The V3–V4 hypervariable regions of the *16S* rRNA were amplified in this study, which is also used for microbiome surveys in ticks [16, 47] and in spider mites [55]. An earlier study by Sperling et al. [56] found that V4 amplicons can identify more bacteria in tick microbiome surveys.

Different analyses demonstrated that the *D. marginatus* and *D. reticulatus* microbiomes differ in their diversity and taxonomic structure. Furthermore, species- and sex-specific bacteria were detected from *D. marginatus* and *D. reticulatus*. In detail, *D. reticulatus* harbored more bacteria than *D. marginatus*, and the bacterial diversity in tick males seemed higher. The PcoA results suggested that the same species or sex have similar microbiome compositions. In addition, bacterial relative abundance differed between species and sexes, and specific bacteria were generally prevalent in their tick hosts. This study provides further evidence that host-related factors affect tick microbiome diversity and composition. Previous studies have revealed that the tick microbiome could vary depending on other factors, such as the season during which ticks were collected [57], geographical region [51, 58], tick developmental stages and tissues [16, 50, 58, 59], tick feeding status [60, 61] and presence of pathogens [17, 50].

Proteobacteria were the most abundant phylum in the two species and the phyla Actinobacteria, Bacteroidetes, Firmicutes and Tenericutes had high relative abundance; these findings are consistent with the findings in other tick species [60, 62, 63]. A special case was found in *D. marginatus* males, which had high relative abundance of Chlamydiae. These bacteria were further assigned to the order Chlamydiales, family Rhabdochlamydiaceae and “*Candidatus* Rhabdochlamydia”. Their *16S* rRNA gene sequences were similar to “*Candidatus* Rhabdochlamydia porcellionis”, a known intracellular pathogen from the hepatopancreas of the terrestrial isopod *Porcellio scaber* [64]. Rhabdochlamydiaceae was also present in other arthropods, such as cockroaches **[**65**]** and dwarf spiders [66]. In ticks, Rhabdochlamydiaceae was identified in *I. ricinus* [67, 68] and *Hyalomma dromedarii *[67]. These observations suggest that arthropods can be reservoirs and vectors of the Rhabdochlamydiaceae. The pathogenic roles of Rhabdochlamydiaceae are not clear, mainly due to the almost complete absence of diagnostic tools and the difficulties encountered in attempts to cultivate Rhabdochlamydiaceae. Considering the fact that ticks can transmit some bacteria of Chlamydiales to humans and animals [69], investigating the prevalence of Chlamydiales within wild and farm animals, as well as the prevalence in humans with and without a history of tick bites, is necessary in the future.

As an obligate intracellular bacteria associated with ticks, *Rickettsia* can be divided into pathogens and non-pathogenic symbionts [70]. In the present study, *Rickettsia* has been shown to be prevalent in both *D. marginatus* and *D. reticulatus*. The *16S* rRNA gene fragments used for amplification are highly conserved within *Rickettsia*, which hinders their species-level identification. Similar patterns of *Rickettsia* infection were found in *D. marginatus* studied in Turkey [48] and *D. reticulatus* studied in Russia [47]. Additionally, Duron et al. [19] found that *Rickettsia*-like endosymbionts are common in various tick species, including *D. marginatus*. The effects of non-pathogenic *Rickettsia* spp. on tick biology are poorly understood. *D. marginatus* and *D. reticulatus* are widely distributed across Europe and known as vectors of two pathogenic *Rickettsia* (*R. slovaca* and *R. raoultii*) [34, 71, 72]. Therefore, further efforts are needed to distinguish if *Rickettsia* are pathogenic or non-pathogenic endosymbionts and to explore their biological effects.

Besides the high prevalence of *Rickettsia*, some soil or environmental bacterial genera such as *Brevibacterium*, *Pseudomonas*, *Sphingomonas* and *Rhodococcus* were abundant in the two species of tick. These bacteria were also detected in many other tick species, although sterilization has been performed prior to DNA isolation [16, 24, 59, 62, 63]. This may be due to inadequate sterilization, or that these bacteria may have been ingested by ticks during feeding and therefore present in the tick midgut [14, 16]. Studies in nymphal and adult *I. scapularis* provided supportive evidence, as both dissected gut tissues and whole ticks showed many common genera such as *Stenotrophomonas*, *Sphingobacterium*, *Pseudomonas* and *Acinetobacter*, suggesting that these bacteria are likely *bona fide* tick gut residents [24, 59].

At least ten maternally inherited bacteria have been found in ticks [19]. Among them, five of six observed bacteria showed a specific association to tick species in this study. An earlier study by Duron et al. [19] revealed the presence of *Coxiella*, *Rickettsia* and *Spiroplasma* in *D. marginatus*, and the presence of *Francisella* in *D. reticulatus*. NGS analysis also found that Russian *D. reticulatus* harboured *Francisella* [47] whereas in another study of the bacterial infections of *D. reticulatus* in Slovakia, *R. raoultii*, *R. slovaca*, *Coxiella burnetii*, *Coxiella*-like and *Francisella*-like endosymbionts were detected [37]. NGS analysis of *D. marginatus* in Turkey only found *Rickettsia* [48]. These findings further suggest that the bacterial compositions in the two species are influenced by their geographical distribution. Tick sex is another factor influencing bacterial infections, as females and males had different bacterial abundance [47, 48]. The roles of most bacteria have yet to be clearly elucidated [18]. However, the essential roles of *Coxiella*-like and *Francisella*-like endosymbionts have been reported in several tick species, in which these bacteria may provide essential nutrients for the ticks [20–23]. Given the high prevalence of *Coxiella*-like and *Francisella*-like endosymbionts in *D. marginatus* and *D. reticulatus*, further studies examining the mutualistic relationships between these endosymbionts and their tick hosts are warranted. In addition, *Spiroplasma* and *Arsenophonus* were abundant in *D. marginatus*. Their presence in different tick species have also been summarized [73]. *Spiroplasma* and *Arsenophonus* act as male-killers in some other arthropod species [74, 75]. However, no male-killing effect was observed in *D. marginatus*, even though they were detected in males.

## Conclusions

The bacterial microbiomes of field-collected *D. marginatus* and *D. reticulatus* from Slovakia differed in their diversity and taxonomic structure. Tick phylogeny and sex were two factors influencing the bacterial microbiome. In detail, *D. reticulatus* harbored more bacteria than *D. marginatus*, and the bacterial diversity in tick males seemed higher. A possible bacterial pathogen “*Candidatus* Rhabdochlamydia sp.” was detected from *D. marginatus* males. *Rickettsia* was the most abundant and other maternally inherited bacteria also had high relative abundance, although their biological roles are unclear. The occurrence of soil or environmental bacterial genera indicated that they may have been ingested by ticks during feeding. These findings will aid in the control of ticks and TBDs.

## Additional files

**Additional file 1: Table S1.** Analysis of the obtained MiSeq sequencing reads per sample.

**Additional file 2: Figure S1.** Rarefaction curves for Shannonʼs index at OTU level.

**Additional file 3: Table S2.** Analysis of taxonomy and abundance of the obtained OTUs.

## Data Availability

All MiSeq sequencing data are available on the NCBI Sequence Read Archive (SRA) under the accession number PRJNA548395.

## Abbreviations

TBDs: tick-borne diseases
HGA: human granulocytic anaplasmosis
TBPs: tick-borne pathogens
NGS: high-throughput next-generation sequencing
*16S* rRNA: *16S* ribosomal RNA
OUT: operational taxonomic unit
