# Supplementary figures and images for: The bacterial microbiome of field-collected Dermacentor marginatus and Dermacentor reticulatus from Slovakia
Source: Parasit Vectors. 2019 Jun 27;12:325. doi: 10.1186/s13071-019-3582-9 (PMC6598266; doi:10.1186/s13071-019-3582-9)

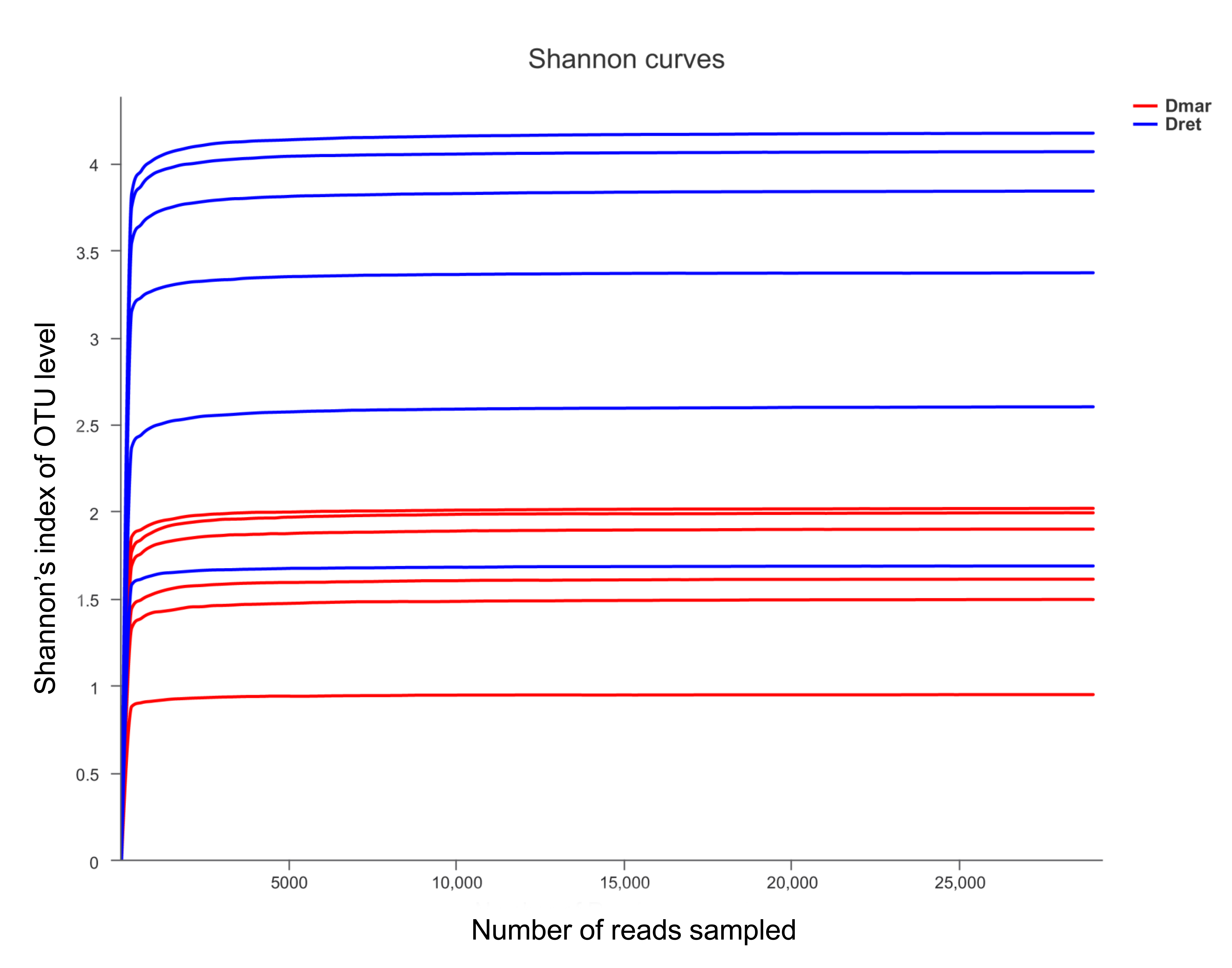

Supplement: Supplementary file 2 — Additional file 2: Figure S1. Rarefaction curves for Shannonʼs index at OTU level. [file 13071_2019_3582_MOESM2_ESM.tif]
